# Relationship Between Subclinical Mastitis Occurrence and Pathogen Prevalence in Two Different Automatic Milking Systems

**DOI:** 10.3390/ani15060776

**Published:** 2025-03-09

**Authors:** Karise Fernanda Nogara, Marcos Busanello, Maity Zopollatto

**Affiliations:** Department of Animal Science, Federal University of Paraná, Curitiba 80035-050, PR, Brazil; marcosbusanello@hotmail.com (M.B.); maity@ufpr.br (M.Z.)

**Keywords:** contagious pathogens, dairy cows, robots in agriculture, teat-cleaning process

## Abstract

This study compared two types of automatic milking systems (AMSs) and their relationship with epidemiological indices of subclinical mastitis (SCM) and prevalence of mastitis-causing pathogens. Conducted between 2020 and 2023 on a dairy farm in Vacaria, Rio Grande do Sul, Brazil, this study analyzed data from 464 lactating cows housed in compost-bedded pack barns (CBPBs) and milked by eight AMS units: four from DeLaval (which utilizes teat cup for teat cleaning) and four from Lely (which utilizes brushes for teat cleaning). SCM incidence, prevalence, percentage of chronic, and cured cows were determined using somatic cell counts (SCCs) and microbiological cultures. Statistical analyses included the Wilcoxon signed-rank test and Chi-square test to evaluate SCM indices and pathogen associations with AMSs. No significant difference was observed in SCM prevalence (*p* = 0.3371), percentage of chronic (*p* = 0.3590) and cured cows (*p* = 0.4038), SCC (*p* = 0.1290), and total bacterial count (TBC) (*p* = 0.8750) between AMS types. However, the SCM incidence was higher in the Lely (14.7%) than in the DeLaval AMS (9.1%) (*p* = 0.0032). The Chi-square results revealed that the Lely AMS was associated with major pathogens like *Staphylococcus aureus* and *Escherichia coli*, whereas DeLaval showed associations with minor environmental and contagious pathogens, particularly non-aureus *Staphylococci*. The findings indicate a relationship between AMS-cleaning systems and pathogen spread, suggesting that Lely AMS may contribute to more aggressive infections due to its cleaning system.

## Simple Summary

The utilization of automated milking systems (AMSs) has grown significantly worldwide, resulting in higher productivity and reduced demand for manual labor. However, important differences have been observed in relation to the health of the mammary gland of dairy cows, especially with regard to mastitis. Based on the analysis of a database from a dairy farm, it was found that cows milked by the Lely AMS had a higher incidence of mastitis caused by primary contagious and environmental pathogens, the main pathogens responsible for compromising the health of the mammary gland. On the other hand, in the DeLaval AMS, mastitis was predominantly associated with secondary pathogens, including both contagious and environmental pathogens. These results suggest that differences in AMS may influence the predisposition to contamination in dairy cows, possibly due to variations in the cleaning methods adopted by each system.

## 1. Introduction

In 2021, Rio Grande do Sul ranked as the third highest milk-yielding state in Brazil [1]. However, 61% of the dairy farms in this region closed their operations between 2015 and 2023 [1]. The main reasons were a scarcity of specialized labor, lack of family succession, and rising production costs [1]. This scenario led farmers to adopt automatic milking systems (AMSs) to reduce labor requirements and remain in dairy farming [2]. Nowadays, the Rio Grande do Sul and Paraná state are the leaders in the adoption of AMS in Brazil [2].

Although AMS technology is well-established worldwide and its adoption among Brazilian farmers is growing, many still have concerns about its financial viability and its impact on animal health and milk quality. However, research on AMS in the South American and Brazilian context is still limited. In this sense, Morales-Piñeyrúa et al. [3] evaluated cow temperament and milking performance during the adaptation period to an AMS in a Uruguayan scenario, while Rodriguez et al. [4] conducted a comparative analysis of milking and behavioral characteristics in multiparous and primiparous cows milked in AMSs in Brazil. Other Brazilian studies have focused on correlating milking characteristics and cow behavior in AMS [5] and on the impact of animal welfare and productive factors on the AMS-milking frequency [6].

Research on cow health issues in AMSs in South America is limited. One notable study is by Miguel-Pacheco et al. [7], which evaluated behavioral changes in lame cows milked using AMSs at Chile. However, one of the key concerns regarding the health of cows milked via AMSs is the monitoring of mastitis. This is particularly relevant to the teat-cleaning process, which is entirely performed by the robot without human supervision [8,9,10]. The AMS technology differs in its approach to teat cleaning. Manufacturers like DeLaval International A.B. (Tumba, Sweden) (VMS models), BouMatic Robotics (Emmeloord, The Netherlands) (MR-S2 and MR-D2 models), S.A. Christensen & Co (Kolding, Denmark) (RDS FutureLine model), and Insentec (Marknesse, The Netherlands) (Galaxy Starline model) utilize a separate cleaning cup for teat cleaning, whereas Lely International N.V. (Maassluis, The Netherlands) (Astronaut models) and Lemmer Fulwood (Ellesmere, England) (M^2^erlin model) utilize brushes, and GEA WestfaliaSurge (Oelde, Germany) (MIone model) utilizes the same milking teat cup for both milking and cleaning [11]. Scientific literature reports evidence that a better teat-cleaning score is associated with the utilization of a cleaning cup rather than brushes for dirty teats before the cleaning process [8]. However, a well-done cleaning of teat cleaning in AMS is dependent on herd and cow characteristics [8,9].

An inadequate teat cleaning process by the robot can lead to both an increase in the total bacterial count (TBC) in milk and the occurrence of mastitis [12]. Mastitis is the main disease in dairy cattle. In Brazil, the prevalence and incidence of subclinical mastitis (SCM) is around 46% and 17%, respectively [13]. To control those indexes, the teat cleaning process can be a key factor in reducing the spread of mastitis-causing pathogens in AMSs [8,10], especially contagious pathogens like *Staphylococcus aureus* [14]. Mastitis detection in AMS is based on various variables, such as the electrical conductivity of milk, milk coloration, the presence of blood in milk, milk yield by mammary quarter, somatic cell count (SCC), among others, which vary by manufacturer [15,16]. Those variables jointly generate an alert from the system, signaling to the farmer a possible milk alteration that could indicate a new intramammary infection (IMI) in a cow [16].

While most studies focus on clinical mastitis (CM) occurrence and diagnosis in AMSs [16,17,18], Hiitiö et al. [19] is one of the few studies to address the occurrence of SCM in AMSs. Some studies have examined the prevalence of mastitis-causing pathogens in AMSs [20,21] and their potential spread through the system [22]. However, there are still limited data on this topic, especially in Brazil and South America.

So, our research aims to evaluate the prevalence and microbiological profile of mastitis-causing pathogens in two types of AMSs: one using brushes and the other using a separate cleaning cup for teat cleaning. This evaluation considers epidemiological indices of SCM (prevalence, incidence, chronic, and cured cows) and milk quality parameters (somatic cell count—SCC and total bacterial count—TBC). Based on previous research, our hypothesis is that the AMS using brush cleaning for teat preparation may have poorer epidemiological indices and lower milk quality due to a higher spread of mastitis-causing pathogens.

## 2. Materials and Methods

### 2.1. Study Design, Dataset, and Farm Characteristics

This research was designed as a longitudinal retrospective study and followed the STROBE statement guidelines for reporting results [23]. The data were provided by a dairy farm from Vacaria county, Rio Grande do Sul State, Brazil, located at latitude 28°30′39″, longitude 50°55′47″, and an altitude of 971 m. The region has a Cfb-type climate with temperate summers [24]. Data were collected from March 2020 to July 2023 from two separate compost-bedded pack barns (CBPBs): one containing four DeLaval AMSs that utilize teat cups for teat cleaning (V300 model; DeLaval International A.B., Tumba, Sweden) and the other containing four Lely AMSs that utilize brushes for teat cleaning (two Astronaut A4 and two Astronaut A5 models; Lely International N.V., Maassluis, The Netherlands).

The CBPB beddings were composed of a mixture of sawdust and shavings and were turned twice a day with a scarifier and a rotary hoe to break up aggregates. The materials were replaced as necessary when it was observed that the bed did not reach the ideal temperatures of 45 to 60 °C. The CBPB with DeLaval AMSs presented more challenges in maintaining the bedding temperature at ideal levels, with high humidity and the presence of clods. Due to the guided-flow traffic design (milk-first), certain areas of the bedding had lower quality, with small elevations and holes caused by increased herd movement, particularly near access and exit gates. The installation with Lely AMSs (free cow traffic design) presented a slightly drier bedding in comparison with the other CBPB; however, few points of the bedding were at ideal temperature. That barn had problems with rainwater entering the sides due to the short eaves.

The DeLaval AMS robotic arms are designed to locate teats and udders of various shapes using camera-based mapping technology. In addition to attaching teat cups, these robotic arms perform essential tasks such as cleaning, preparing teats, and applying disinfectants via dual spray nozzles [25]. The DeLaval AMS system utilizes a circular teat-cleaning methodology that combines warm water, a cleaning solution, and air, integrated with a specialized disinfection cup. This cup facilitates the drying of teats post-cleaning and the removal of foremilk [26,27,28], which is diverted to a separate line to reduce the risk of cross-contamination.

After each milking session, the V300 system (DeLaval AMS) executes a self-cleaning cycle, which encompasses the cleaning of the chamber, comprehensive washing of teat cups and hoses (both internally and externally), and the option for complete disinfection of all four milking cups and the hygiene cup. The cleaning parameters can be customized to align with the hygiene conditions of the cows’ housing environment. During the milking process, the DeLaval AMS collects detailed data, including production and milk flow from each quarter, milking interval, presence of blood, milk electrical conductivity, and other critical parameters. Milk is directed to the cooling tank only after analysis for blood presence, milk electrical conductivity, and the mastitis detection index [25].

The Lely AMS features a flexible arm positioned beneath the cow, designed to prevent teat cups from falling to the ground and to enhance control during the milking process. Teat detection and handling are facilitated by a 3D camera system [29]. For teat cleaning, the AMS employs double rotating brushes, which also remove the foremilk within the same milking flow cup [22,30]. The cleaning process lasts approximately 45 s per cow, depending on the programmed schedule [27]. To minimize cross-contamination, the brushes are disinfected using a chlorine-free detergent.

The Lely AMS milking units undergo three automated cleaning cycles daily, which include washing and disinfecting the milking cups, cleaning units, milk collection tank, and both short and long milking tubes. Additionally, the brushes are sprayed with disinfectants as part of the process [22]. The milking unit is sanitized using hot steam at 150 °C, eliminating the need for detergents [29]. The system is equipped with tools for real-time milk quality assessment during milking. Parameters such as fat, protein, and lactose concentrations are measured, alongside mastitis indicators (milk color, electrical conductivity, and SCC). It also monitors cow health metrics, including rumination time, activity levels, weight, feed intake, and leftover’s feed, as well as other performance indicators like milk yield, milking duration, flow rate for each mammary quarter, and milk temperature [29]. The disinfection of the teat after milking, in both AMSs (Lely and DeLaval), is realized with iodine jets.

### 2.2. Cows

The herd had, on average, 460 Holstein cows, evenly distributed into two barns with 230 cows each. Within each barn, approximately 92 cows (40%) were primiparous, and 138 cows (60%) were multiparous, being milked on these AMSs each month, averaging 55 to 60 cows per AMS. The cows had a median age of three years old and 700 kg of body weight.

The cows’ diets remained consistent in terms of ingredients but varied in composition throughout the study. Details of the feed provided in the feeding lane, measured on a natural matter basis, are presented in Table 1. The quantity of feed offered in the robot box was adjusted according to milk production. For cows producing an average of 40 L of milk per day, approximately 5.6 kg of feed was provided. The herd’s estimated dry matter intake was 27 kg/day. On average, the Lely batches produced 42 L/cow/day, while the DeLaval batches averaged 44 L/cow/day. Robot access permissions were determined by the cows’ days in milk and gestation stage.

The animals were regularly hoof trimming on the farm to promote greater locomotor comfort. In addition, preventive management practices, such as flame singeing, were routinely performed to reduce manure accumulation, keep the animals cleaner, improve udder health, and facilitate teat detection by the robot. Most animals had poor udder and teat conformation, which often made it difficult for the AMSs to connect the teat cups. However, no data were available on the animals’ hygiene score, as the farm did not perform this assessment regularly, and the information analyzed came from an existing database.

Data relating to the tank’s TBC and SCC were provided by the farm, through an official analysis carried out by the dairy company that buys the raw material. In the farm were two milk bulk tanks, one for DeLaval AMSs and the other for Lely AMSs. Thus, the milk of both was not mixed.

### 2.3. Epidemiological Indexes

The individual SCC was obtained monthly from the official dairy control service to calculate the prevalence and incidence of SCM, as well as the percentage of chronic and cured cows. A SCM case was considered when cows had an SCC of ≥200 × 10^3^ cells/mL [31]. The SCM prevalence was defined as the number of cows with a SCC ≥ 200 × 10^3^ cells/mL divided by the total number of tested cows on a given test day [13], in this case monthly. The incidence of SCM was defined as the number of cows whose SCC increased from <200 × 10^3^ cells/mL to ≥200 × 10^3^ cells/mL on two consecutive test days, divided by the number of cows whose SCC was <200 × 10^3^ cells/mL on the previous test day [32]. Chronic cases were defined as the number of infected cows (SCC > 200 × 10^3^ cells/mL) in two consecutive test days divided by the total of tested cows. The proportion of cured cows was defined as the percentage of cows with SCC > 200 × 10^3^ cells/mL on a previous test day that dropped to <200 × 10^3^ cells/mL on the current test day, calculated as the number of cured cows divided by the total number of cows with SCC > 200 × 10^3^ cells/mL on the previous test day.

### 2.4. Microbiological Culture

**Figure 1:**
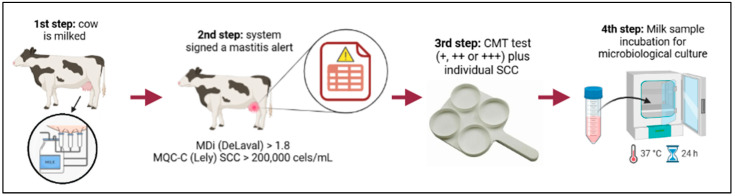
Scheme illustrating the process utilized to determine whether a milk sample from a cow should be sent for microbial culture. The sequence of steps was as follows: (1) Cow was milked, (2) a mastitis alert was signaled by the system (DeLaval AMS MDi > 1.8; Lely AMS MQC-C based on SCC > 200 × 10^3^ cells/mL [16]), (3) quarter samples were submitted to CMT test plus cow’s individual SCC analysis, and (4) mixed-milk sample was incubated for microbiological culture.

Each AMS has its own system and indicators; for example, Lely AMS utilizes the MQC-C (Milk Quality Check—Cows) indicator to issue alerts related to milk color, electrical conductivity per mammary quarter, milk fat/protein index, milk lactose index, temperature, SCC, and more [16]. The DeLaval AMS utilizes the Mastitis Detection Index (MDi), which is characterized by a combination of milk electrical conductivity, milking interval, blood in milk, and SCC. When alerts were issued, farm veterinarians inspected the animals [16]. To confirm SCM, veterinarians evaluated the reaction to the CMT test. To confirm clinical mastitis, veterinarians checked for any visible changes in the milk, such as lumps or blood. In positive cases identified by these methods, milk samples were collected to determine the causative agent of mastitis by microbiological culture. Microbiological cultures were conducted using on-farm microbiological culture by OnFarm^®^ (Piracicaba, Brazil). OnFarm^®^ utilizes a chromogenic culture medium in which samples are incubated at 37 °C for 24 h, enabling the identification of mastitis-causing pathogens within this period, according to Garcia et al. [33]. Figure 1 illustrates the sequential process used to determine whether a milk sample from a cow should be sent for microbial culture or not.

Currently, with the utilization of artificial intelligence (AI), data interpretation is performed using applications that color-analyze images of the plates, categorizing them as positive or negative [33]. In positive samples, the causative agent of mastitis was identified, and AI indicated the need for antimicrobial treatment, in addition to recommending the duration and appropriate active ingredient. This approach made the utilization of antibiotics more assertive, especially considering pathogens such as *Escherichia coli*, which, in many cases, do not require therapeutic intervention due to the possibility of spontaneous cure [34,35].

### 2.5. Statistical Analysis

Since SCC and TBC did not exhibit residual normality, we utilized the Wilcoxon signed-rank test, with the month considered a paired sample to compare the two different AMS. The same test was applied to SCM prevalence, incidence, and proportion of chronic and cured cows to standardize the statistical procedure. Cohen’s *r* was utilized as a non-parametric measure of effect size [36].

Microbiological culture data were analyzed in two steps. First, pathogens were grouped as major contagious (*Mycoplasma bovis, Staphylococcus aureus, Streptococcus agalactiae,* and *Streptococcus dysgalactiae*), major environmental (*Escherichia coli, Klebsiella* spp., and *Streptococcus uberis*), minor contagious (*Corynebacterium bovis* and non-aureus *staphylococci*), and minor environmental ones (other pathogens including *Bacillus* spp., *Enterobacter* spp., *Enterococcus* spp., *Lactococcus* spp., *Nocardia* spp., *Prototheca* spp., *Pseudomonas* spp., other *Streptococci* species, and yeasts), as described by Cobirka et al. [14]. So, a Chi-square test was applied to verify the association between mastitis-causing pathogen groups and AMSs. In the second step, the association was examined based on the prevalence of individual pathogens with respect to the AMSs. When associations were found to be significant, the null hypothesis that the row and column variables are independent was further tested using the studentized residual. This residual is the ratio of the difference between the observed frequency and the expected frequency to its standard error, where the observed frequency is the count in the table cell and the expected frequency is derived from the Chi-square test [37].

All analyses were performed using SAS OnDemand, version 3.81 [38]. The Wilcoxon signed-rank test was conducted with SAS PROC UNIVARIATE, and Chi-square tests were carried out using SAS PROC FREQ. Data for SCC, TBC, and SCM indices (prevalence, incidence, % of chronic, and cured cows) are shown in boxplots with their respective medians, while microbiological data are presented as frequencies. The significance level was set at 5%.

## 3. Results

**Figure 2:**
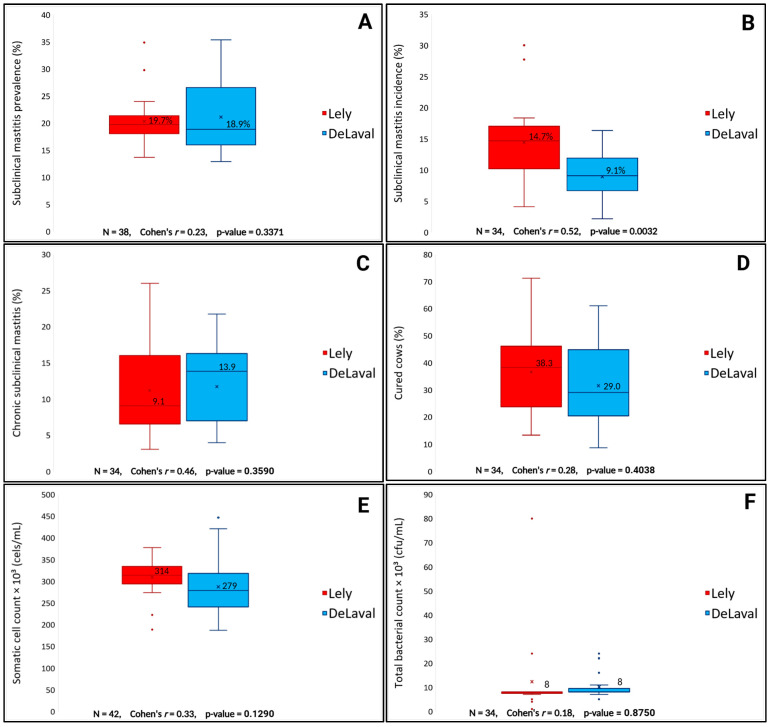
Median comparisons from the Wilcoxon signed-rank test for subclinical mastitis prevalence (**A**), incidence (**B**), percentage of cows with chronic subclinical mastitis (**C**), and cured cows (**D**), bulk tank milk somatic cell count (**E**), total bacterial count (**F**) between Lely and DeLaval AMSs.

The SCM prevalence did not differ between AMSs (*p* = 0.3371), being 19.7% for the Lely AMS and 18.9% for the DeLaval AMS (Figure 2A). In contrast, the SCM incidence differed between the AMSs (*p* = 0.0032), with the Lely AMS showing a 14.7% incidence compared to 9.1% for the DeLaval AMS (Figure 2B). The percentage of chronic cows (Lely = 9.1%, DeLaval = 13.9%, *p* = 0.3590, Figure 2C) and cured cows (Lely = 38.3%, DeLaval = 29.0%, *p* = 0.4038, Figure 2D) was also not significant. Bulk tank milk SCC (*p* = 0.1290, Figure 2E) and TBC (*p* = 0.8750, Figure 2F) did not differ significantly between Lely AMS (314 × 10^3^ cells/mL and 8 CFU/mL, respectively) and DeLaval AMS (279 × 10^3^ cells/mL and 8 CFU/mL, respectively).

A total of 1500 composite milk samples from cows with AMS alerts, positive CMT test, and SCC > 200 × 10^3^ cells/mL were incubated on plates for microbiological analysis (Lely AMS: N = 951 [63.4%], DeLaval AMS: N = 549 [36.6%]). Of these, 630 (42%) milk samples resulted in no growth (Lely AMS: N = 406 [64.4%], DeLaval AMS: N = 224 [35.6%]), while the remaining 870 (58%) milk samples resulted in growth of some pathogen (Lely AMS: N = 545 [62.6%], DeLaval AMS: N = 325 [37.4%]).

For the samples that showed growth on plates, the majority of SCM cases were caused by minor contagious pathogens (N = 521 [59.9%]), followed by major contagious pathogens (N = 160 [18.4%]), major environmental pathogens (N = 142 [16.3%]), and minor environmental pathogens (N = 47 [5.4%]) (Table 2). A significant association was found between groups of mastitis-causing pathogens and AMS (χ^2^ = 28.7; *p* < 0.0001) (Table 2). Major contagious pathogens showed a higher proportion in the Lely AMS (N = 126 [23.1%]) compared to the DeLaval AMS (N = 34 [10.5%]) (Table 2). In contrast, minor contagious (DeLaval AMS: N = 221 [68.0%]; Lely AMS: N = 300 [55.0%]) and minor environmental pathogens (DeLaval AMS: N = 24 [7.4%]; Lely AMS: N = 23 [4.2%]) showed a higher proportion in DeLaval AMS compared to Lely AMS (Table 2).

When examining specific mastitis-causing species of pathogens identified through plate growth, we found that the most prevalent species on that farm were *Staphylococcus chromogenes* (N = 194 [22.3%]), *Staphylococcus aureus* (N = 131 [15.1%]), *Staphylococcus haemolyticus* (N = 106 [12.2%]), *Staphylococcus warneri* (N = 85 [9.8%]), *Klebsiella* spp. (N = 78 [9.0%]), *Escherichia coli* (N = 57 [6.6%]), *Corynebacterium bovis* (N = 47 [5.4%]), and other non-aureus *staphylococci* (N = 89 [10.2%]) (Table 3). A significant association was found between specific mastitis-causing species of pathogens and AMS (χ^2^ = 75.7; *p* < 0.0001) (Table 3). *Staphylococcus aureus* (Lely AMS: N = 106 [19.4%]; DeLaval AMS: N = 25 [7.7%]), *Escherichia coli* (Lely AMS: N = 45 [8.3%]; DeLaval AMS: N = 12 [3.7%]), and *Corynebacterium bovis* (Lely AMS: N = 39 [7.2%]; DeLaval AMS: N = 8 [2.5%]) showed a higher proportion in Lely AMS compared to DeLaval AMS (Table 3). In contrast, other non-aureus *staphylococci* (DeLaval AMS: N = 56 [17.2%]; Lely AMS: N = 33 [6.1%]) and other pathogens (DeLaval AMS: N = 21 [6.5%]; Lely AMS: N = 12 [2.2%]) showed a higher proportion in DeLaval AMS compared to Lely AMS (Table 3).

## 4. Discussion

We did not find differences for SCC, TBC, prevalence of SCM, and percentage of chronic and cured among the AMSs evaluated. On the other hand, the incidence of SCM and the primary pathogens causing contagious mastitis were more commonly associated with Lely AMS, while environmental and secondary contagious pathogens were more associated with DeLaval AMS.

The effects observed on SCM incidence, but not prevalence, can be attributed to the following factors: (a) bacterial biofilm formation, (b) the performance of on-farm microbiological culture, and (c) the possible difference in milking frequency between AMSs. Bacteria such as *Staphylococcus aureus* (low cure rate) and *Escherichia coli* (high potential for spontaneous cure), which were frequently associated with the Lely AMS, are capable of biofilm formation [39,40]. This protective structure promotes bacterial proliferation and increases resistance to disinfectants, which may have contributed to the high incidence of SCM in Lely AMS. Another relevant factor is the farm’s management strategy, which utilizes OnFarm^®^ technology for rapid detection (within 24 h) of positive samples and identification of mastitis-causing pathogens [33]. This enables more precise and targeted treatment, reducing the indiscriminate utilization of antibiotics, particularly in cases of mastitis caused by *Escherichia coli*, which often resolves spontaneously [34,35]. Finally, the milking frequency on the AMS may also play a role because in guided-flow traffic designs, cows typically have more frequent milkings, which can contribute to improving teat hygiene by reducing the bacterial load in the teat canal and promoting keratin renewal [10].

Although not significant, the proportion of cured cows was 9% higher in the Lely AMS, potentially contributing to balancing SCM prevalence between the AMSs. Another possible influence variable, but not included in this study (data not provided by the farm), is the number of visits. With AMSs, the higher milking frequency reduces udder pressure and the colonization time of mastitis-causing pathogens. However, AMSs can also serve as a vehicle for the transmission of bacteria, especially contagious ones, from sick to healthy cows [41,42]. Conversely, long periods between cow visits can allow warm milk to remain in the lines, as well as drying films on surfaces [43].

The first place where milk is contaminated outside the cow is the milking equipment [43]. As mentioned earlier, both the milking equipment and the operating process are different between brands on the market. The DeLaval AMS employs an exclusive teat cleaning cup for cleaning and removing the first three milk jets. In contrast, Lely AMS utilizes teat cleaning brushes and removes the first three milk jets in the same teat cup, diverting it to the drain. Cleaning with exclusive cups is preferable to brushes, especially for very dirty teats [8]. Brushes can clean the base of the udder, but the water utilized can run into the teat cup, increasing the bacterial load and the risk of mastitis [44]. We hypothesize that residual contamination may be present in the Lely AMS system.

The goal of teat cleaning in AMS is to minimize the transfer of mastitis-causing bacteria [26,45] by removing the first milk jets, disinfecting, cleaning, and drying the teats to ensure high-quality milk and stimulate milk ejection [46]. However, the effectiveness of cleaning methods can be compromised by technical failures, such as problems with teat cup positioning, teat cleaning, and brush maintenance [10,47]. These issues can impact teat bacterial counts and cow restlessness (cows may move after the teat is positioned), long udder hair, as well as abnormal udder and teat structure [8]. Moreover, the robot cannot distinguish between a clean and a dirty teat before automated cleaning [18,41]. Irregular, deep, and shallow udders tend to receive less effective cleaning compared to normal udders when using brushes for hygiene (as in Lely AMS) [8]. Normal udders, above the hock, exhibit satisfactory mammary gland conformation and health, with lower SCC and bacterial counts in the teats. Cows with shallower udders seemed to have better teat-cleaning effectiveness [48]. Therefore, udder depth, in addition to affecting milk production and quality, also influenced the degree of teat dirtiness. Cows with deep udders present a higher risk of IMI and chronic infections, while cows with dirty udders have a higher risk of new cases of SCM [49]. Córdova et al. [48] found that the depth of the udder influences the dirtiness of the teats, since deep udders are less effective in cleaning and sanitizing the teats, explaining 70% of their data variability.

All AMSs include an opportunity for post-milking teat disinfection with iodine, but their reliability in adequately covering teats is debatable [50]. The iodine pre-dip procedure (0.1–0.25%) aims to reduce IMI with environmental pathogens [45]. The proportion of teats not covered with iodine spray has been associated with new infections and cows with high SCC in AMS, being that in 18% of milkings teats were not completely covered with iodine [9]. Post-milking teat disinfection is one of the most important hygienic measures to control the spread of pathogens, eliminating any bacteria that may colonize the teats [38,51], which consequently cause SCM [52]. This management plays a fundamental role in milk yield, especially in early lactation, a critical transition period in which cows are immunosuppressed. This effect is particularly evident in multiparous cows, which have reduced neutrophil activity, making them more susceptible to intramammary infections. In contrast, primiparous cows have higher neutrophil functionality, which may contribute to a lower severity of coliform mastitis [53].

The milking unit can act as a source of cross-contamination, transferring bacteria to cows, mainly contagious bacteria, and can cause new IMI [22,50] due to discrepancies in cleaning methods between manufacturers and compared to conventional milking [43,48,50]. To reduce this problem, cleaning of milking equipment in AMSs can be performed two to three times a day [26], with possible adjustments as needed. During critical periods, when cows arrive dirtier for milking, the cleaning settings of DeLaval AMS can be adjusted for greater effectiveness. However, changes in the number of cleaning cycles (increase or decrease) can impact the robot’s operating time and, consequently, its efficiency.

On farms with AMS, there is also a relationship between high SCC and the proportion of cows with dirty teats before milking [9], whereas pathogenic agents such as *Klebsiella* spp. may be associated with the hygiene of the cow and udder [54,55]. The increase in milk SCC depends on the pathogen causing the inflammation, i.e., its pathogenicity, as *Corynebacterium bovis* did not increase milk SCC, unlike primary pathogens such as *Staphylococcus aureus*, *Streptococcus agalactiae*, and *Streptococcus uberis* [56]. Coliforms are indicators of environmental contamination and may be associated with the hygiene of animal housing and during milking [26]. Low efficiency in teat cleaning favors higher coliform counts in milk [57]. However, the distribution of mastitis pathogens may be associated not only with housing conditions but also with the milking system [20]. Teat cleanliness before entering the milking box is as important as the cleaning performed by the robot. This underscores the significance of maintaining bedding conditions and cleaning resting areas, as the robot alone cannot remove all the dirtiness. Dirty teats before milking result in lower cleaning effectiveness [48,58,59]. However, AMS technology does not allow for the cleaning of only dirty teats or more efficient cleaning of those, as there is no selection criterion. This would be especially necessary during times of the year when environmental conditions hinder hygiene [27]. This underscores the need for pre- and post-milking procedures in AMSs to be performed correctly to minimize the transmission of pathogens among the herd.

The association of secondary contagious and environmental pathogens in the DeLaval AMS may be linked to the CBPB bedding system utilized on the farm. Bedding is an aggravating factor for udder health in animals, especially when appropriate temperatures (45 to 60 °C) are not maintained, hindering the drying process, and consequently increasing the risk of SCM in the herd [60,61]. The bacterial count on the teat, as well as its dirtiness, are reflections of the condition of the environment where the cows are. Cows with a high dirtiness score are directly influenced by the moist condition of the bedding where they are located, especially in CBPB [62]. Bedding materials are sources of microorganisms and may be associated with a higher incidence of mastitis [63]. Both management and cleaning of bedding areas, attention to stocking density, cleaning of feeding alleys, and waiting areas are important to minimize udder dirtiness [10]. Poor hygiene promotes bacterial growth and increases the chance of milk contamination [64]. Teats that were not adequately cleaned during pre-milking favored greater bacterial contamination in the milk [45], highlighting the association between poor teat and udder hygiene and increased new IMI cases [10]. Other risk factors include cows with milk leakage, such as CM and high SCC [50,57], improper milking procedures, insufficient post-milking teat dipping, inadequate housing cleanliness, and poorly designed cow treatment and testing strategies [58,65]. Therefore, in future research, at an experimental level, we suggest evaluating the dirtiness score of animals and bacterial load of the teats before and after AMS cleaning, in order to verify whether the cleaning by the robot is efficient. Therefore, the environment and milking equipment are a potential source of infection for the herd.

The incidence of IMI is highly correlated with the number of mastitis pathogens at the teat end during milking [45], which may lead us to assume that teat cleaning in the Lely AMS may not be performed perfectly. We hypothesize that more disinfection cycles may be necessary in this system; however, further studies are required. Hygiene interventions in herds milked by Lely AMS have shown reductions of 19% in the transmission of *Streptococcus agalactiae* and 17% in *Streptococcus dysgalactiae* when brushes were replaced daily and disinfectant sprays were applied [22]. According to Castro et al. [20], the high prevalence of *Streptococcus dysgalactiae*, *Streptococcus uberis*, and *Staphylococcus aureus* may be associated with less-than-ideal cleaning and disinfection of teat cups, brushes, and milk lines in AMS. Additionally, Vissio et al. [66] found the predominance of *Streptococcus uberis, Corynebacterium* spp., and coagulase-negative *staphylococci* (CNS) in mammary quarters of cows with new IMI when comparing the effectiveness of teat disinfectants in DeLaval AMS. SCM is often caused by secondary pathogens, including coagulase-negative *Staphylococcus* spp. and *Corynebacterium bovis*, although primary pathogens, mainly *Staphylococcus aureus*, also cause it [67]. In AMS, environmental pathogens most associated with SCM and CM were environmental *streptococci* and CNS, while *Staphylococcus aureus* was the contagious pathogen that most contributed to subclinical infections [41]. A low frequency of *Staphylococcus aureus*, a primary pathogen, indicates that the herd is well-managed concerning udder health [66].

Edmondson [52] observed an increase in SCC from 91 × 10^3^ cells/mL to 554 × 10^3^ cells/mL after replacing manual post-milking teat disinfection with an automatic spraying system, with *Staphylococcus aureus* (74%) being the most isolated pathogen. The study suggested that inadequate iodine coverage (10–20%) due to the single iodine jet in the AMS contributed to ineffective disinfection and the spread of intramammary infection. *Staphylococcus aureus*, the most common pathogen causing udder infections in dairy cows [68], is also predominant in AMSs [22], supporting our findings. It primarily resides in the mammary gland and teat skin, causing contagious mastitis, subclinical infections, increased bulk tank SCC, and potentially becoming chronic and difficult to treat, especially in older cows [33,52]. *Actinobacteria* and *Staphylococcus* spp. were the predominant genera found in milk and teat skin samples from dairy cows analyzed for microbiomes, indicating potential mastitis-causing pathogens [67].

Based on our findings, it is recommended to adopt an integrated approach to improve udder health in AMSs, as some infectious and environmental pathogens appear to be more associated with one system than the other. As we have seen, the material and quality of bedding, animal hygiene, and milking equipment influence the cow’s exposure to contamination and predispose to mastitis. Future research should investigate how variations in cleaning methods between different AMS models influence pathogen colonization and milk quality. It is essential to develop and test technologies that allow for automatic detection of dirty teats and adjust the cleaning process according to specific needs. In addition, future research should explore the interaction between housing conditions, bedding management, and the effectiveness of teat hygiene before milking. Integrating advanced technologies with rigorous hygiene practices can significantly contribute to reduce the prevalence of mastitis in the herd and improve the overall health of the animals.

## 5. Conclusions

Our findings reveal differences in microbial dynamics between Lely and DeLaval AMSs. Although SCM incidence (5.6% higher in Lely AMS compared to DeLaval AMS) and bacterial profiles varied, there was no significant impact on key milk quality and animal health indicators. Prevalence of major contagious (case of *Staphylococcus aureus*) and environmental mastitis-causing pathogens (case of *Escherichia coli*) was higher in Lely AMS, while minor contagious pathogens (*Corynebacterium bovis* and non-aureus *staphylococci*) and minor environmental pathogens (including *Bacillus* spp., *Enterobacter* spp., *Enterococcus* spp., *Lactococcus* spp., *Nocardia* spp., *Prototheca* spp., *Pseudomonas* spp., other *Streptococci* species, and yeasts) were more prevalent in DeLaval AMS.

These results reinforce the need for specific management strategies for each AMS, with a greater focus on cleaning cycles in systems with a greater challenge of contagious pathogens and on environmental management practices, such as bedding management, to mitigate risks associated with environmental bacteria.

## Figures and Tables

**Table 1:** Nutritional profile of the diet provided in the feeding lane for primiparous and multiparous cows in both AMSs.

Ingredients ^1^	Multiparous	Primiparous
Corn silage	40.00 kg	30.00 kg
Soybean meal	4.70 kg	3.50 kg
Oat silage	5.00 kg	5.00 kg
Commercial concentrated feed	3.50 kg	3.00 kg

^1^ Based on natural matter.

**Table 2:** Absolute and relative frequencies of mastitis-causing pathogen groups and automatic milking systems from the Chi-square association test.

Pathogens Group ^1^	AMS				Total of Cases	%
	Lely	%	DeLaval	%		
Major contagious	126	23.1 a	34	10.5 b	160	18.4
Minor contagious	300	55.0 b	221	68.0 a	521	59.9
Major environmental	96	17.6	46	14.2	142	16.3
Minor environmental	23	4.2 b	24	7.4 a	47	5.4
Total of cases	545	100.0	325	100	870	100.0

^1^ Adapted from Cobirka et al. [14]: Major contagious: *Mycoplasma bovis*, *Staphylococcus aureus*, *Streptococcus agalactiae*, and *Streptococcus dysgalactiae*. Minor contagious: *Corynebacterium bovis* and non-aureus *Staphylococci*. Major environmental: *Escherichia coli*, *Klebsiella* spp., and *Streptococcus uberis*. Minor environmental: other pathogens including *Bacillus* spp., *Enterobacter* spp., *Enterococcus* spp., *Lactococcus* spp., *Nocardia* spp., *Prototheca* spp., *Pseudomonas* spp., other *Streptococci species*, and yeasts; different letters in the lines indicates statistically significant differences for the cell frequency (χ^2^ = 28.7; *p*-value < 0.0001).

**Table 3:** Absolute and relative frequencies of mastitis-causing pathogen species and automatic milking systems from the Chi-square association test.

Mastitis-Causing Pathogen	AMS			Total of Cases	Relative Frequency %	
	Lely	%	DeLaval	%		
*Staphylococcus chromogenes*	121	22.2	73	22.5	194	22.3
*Staphylococcus aureus*	106	19.4 a	25	7.7 b	131	15.1
*Staphylococcus haemolyticus*	58	10.6	48	14.8	106	12.2
*Staphylococcus warneri*	49	9.0	36	11.1	85	9.8
*Klebsiella* spp.	46	8.4	32	9.8	78	9.0
*Escherichia coli*	45	8.3 a	12	3.7 b	57	6.6
*Corynebacterium bovis*	39	7.2 a	8	2.5 b	47	5.4
*Streptococcus agalactiae*	11	2.0	7	2.2	18	2.1
*Serratia* spp.	11	2.0	3	0.9	14	1.6
*Streptococcus dysgalactiae*	9	1.7	2	0.6	11	1.3
*Streptococcus uberis*	5	0.9	2	0.6	7	0.8
Other non-aureus *Staphylococci* ^1^	33	6.1 b	56	17.2 a	89	10.2
Other pathogens ^2^	12	2.2 b	21	6.5 a	33	3.8
Total	545	100.0	325	100.0	870	100.0

Different letters in the lines indicate statistically significant differences for the cell frequency (χ^2^ = 75.7; *p*-value < 0.0001); ^1^ Other *Staphylococci* species were recorded as non-aureus *Staphylococci* and included *Staphylococcus epidermidis*, *Staphylococcus hyicus*, *Staphylococcus simulans*, *Staphylococcus sciuri*, *Staphylococcus xylosus* and others; ^2^ Other pathogens include *Bacillus* spp., *Enterobacter* spp., *Enterococcus* spp., *Lactococcus* spp., *Nocardia* spp., *Prototheca* spp., *Pseudomonas* spp., other *Streptococci* species, and yeasts.

## Data Availability

Data are contained within the article.

## Abbreviations

The following abbreviations are utilized in this manuscript:

AI	Artificial intelligence
AMS	Automatic milking system
CBPB	Compost bedded pack barn
CM	Clinical mastitis
IMI	Intramammary infection
MDi	Mastitis detection index
SCC	Somatic cell counts
SCM	Subclinical mastitis
TBC	Total bacterial count

## References

[1] Emater (Empresa de Assistência Técnica e Extensão Rural) (2023). Relatório Socioeconômico da Cadeia Produtiva do Leite no RS 2023. Emater/RS, 19 p. https://todoelcampo.com.uy/wp-content/uploads/2024/10/PESQUISA_DO_LEITE_2023.pdf.

[2] Diavão J., Silva A.S., Silvi R.R., Teixeira V.A., Tomich T.R., Paiva C.A.V., Campos M.M., Machado F.S., Ferreira R.E.P., Dórea J.R.R., Bassoi L.H., Bernardi A.C.D.C., Vaz C.M.P., Pires J.L.F., Gebler L., Jorge L.A.D.C., Inamasu R.Y. (2024). Sistemas automatizados de ordenha no Brasil: Panorama e percepções. Agricultura de Precisão: Um Novo Olhar na era Digital.

[3] Morales-Piñeyrúa J.T., Sant’Anna A.C., Banchero G., Damián J.P. (2023). Dairy cows’ temperament and milking performance during the adaptation to an automatic milking system. Animals.

[4] Rodriguez F.A.N., Lopes M.A., Lima A.L.R., Almeida G.A.D., Novo A.L.M., Camargo A.C.D., Barbari M., Brito S.C., Reis E.M.B., Damasceno F.A. (2024). Comparative Analysis of Milking and Behavior Characteristics of Multiparous and Primiparous Cows in Robotic Systems. An. Acad. Bras. Ciênc..

[5] Rodriguez F.A.N., Lopes M.A., Lima A.L.R., De Almeida Júnior G.A., Novo A.L.M., Barbari M., Brito S.C., Bassotto L.C., De Camargo A.C., Nascimento E.F.R. (2023). Correlations between milking characteristics and behavior of cows milked in robotic systems. Semin. Cienc. Agrar..

[6] Córdova H.D.A., Alessio D.R.M., Cardozo L.L., Thaler A. (2018). Impact of the factors of animal production and welfare on robotic milking frequency. Pesqui. Agropecu. Bras..

[7] Miguel-Pacheco G.G., Kaler J., Remnant J., Cheyne L., Abbott C., French A.P., Pridmore T.P., Huxley J.N. (2014). Behavioural changes in dairy cows with lameness in an automatic milking system. Appl. Anim. Behav. Sci..

[8] Hovinen M., Aisla A.M., Pyörälä S. (2005). Visual detection of technical success and effectiveness of teat cleaning in two automatic milking systems. J. Dairy Sci..

[9] Dohmen W., Neijenhuis F., Hogeveen H. (2010). Relationship between udder health and hygiene on farms with an automatic milking system. J. Dairy Sci..

[10] Penry J.F. (2018). Mastitis control in automatic milking systems. Vet. Clin. N. Am. Food Anim. Pract..

[11] Sharipov D.R., Yakimov O.A., Gainullina M.K., Kashaeva A.R., Kamaldinov I.N. (2021). Development of automatic milking systems and their classification. IOP Conf. Ser. Earth Environ. Sci..

[12] Bull C.R., McFarlane N.J.B., Zwiggelaar R., Allen C.J., Mottram T.T. (1996). Inspection of teats by colour image analysis for automatic milking systems. Comput. Electron. Agric..

[13] Busanello M., Rossi R.S., Cassoli L.D., Pantoja J.C.F., Machado P.F. (2017). Estimation of prevalence and incidence of subclinical mastitis in a large population of Brazilian dairy herds. J. Dairy Sci..

[14] Cobirka M., Tancin V., Slama P. (2020). Epidemiology and Classification of Mastitis. Animals.

[15] Hogeveen H., Klaas I.C., Dalen G., Honig H., Zecconi A., Kelton D.F., Mainar M.S. (2021). Novel ways to use sensor data to improve mastitis management. J. Dairy Sci..

[16] Bausewein M., Mansfeld R., Doherr M.G., Harms J., Sorge U.S. (2022). Sensitivity and specificity for the detection of clinical mastitis by automatic milking systems in Bavarian dairy herds. Animals.

[17] Hammer J.F., Morton J.M., Kerrisk K.L. (2012). Quarter-milking-, quarter-, udder-and lactation-level risk factors and indicators for clinical mastitis during lactation in pasture-fed dairy cows managed in an automatic milking system. Aust. Vet. J..

[18] Penry J.F., Crump P.M., Ruegg P.L., Reinemann D.J. (2017). Short communication: Cow- and quarter-level milking indicators and their associations with clinical mastitis in an automatic milking system. J. Dairy Sci..

[19] Hiitiö H., Vakkamäki J., Simojoki H., Autio T., Junnila J., Pelkonen S., Pyörälä S. (2017). Prevalence of subclinical mastitis in Finnish dairy cows: Changes during recent decades and impact of cow and herd factors. Acta Vet. Scand..

[20] Castro A., Pereira J.M., Amiama C., Bueno J. (2015). Mastitis diagnosis in ten Galician dairy herds (NW Spain) with automatic milking systems. SJAR.

[21] Mahmmod Y.S., Klaas I.C., Svennesen L., Pedersen K., Ingmer H. (2018). Communications of *Staphylococcus aureus* and non-aureus *Staphylococcus* species from bovine intramammary infections and teat apex colonization. J. Dairy Sci..

[22] Skarbye A.P., Krogh M.A., Denwood M., Bjerring M., Østergaard S. (2021). Effect of enhanced hygiene on transmission of *Staphylococcus aureus*, *Streptococcus agalactiae*, and *Streptococcus dysgalactiae* in dairy herds with automatic milking systems. J. Dairy Sci..

[23] Sargeant J.M., O’Connor A.M. (2014). Issues of reporting in observational studies in veterinary medicine. Prev. Vet. Med..

[24] Alvares C.A., Stape J.L., Sentelhas P.C., Gonçalves J.L.M., Sparovek G. (2013). Köppen’s climate classification map for Brazil. Meteorol. Z..

[25] DeLaval (2019). DeLaval VMS™ 300: A System Approach. 23p. https://www.delaval.com/globalassets/inriverresources/pdfs/2/2-page-view-vms-series-brochure-online-uk12.pdf.

[26] Salovuo H., Ronkainen P., Heino A. (2005). Introduction of automatic milking system in Finland effect on milk quality. Agric. Food Sci..

[27] Jago J.G., Davis K.L., Copeman P.J., Woolford M.M. (2006). The effect of pre-milking teat-brushing on milk processing time in an automated milking system. J. Dairy Res..

[28] Monov V., Karastoyanov D. Innovations in robotic cow milking systems. Proceedings of the 20th International Conference on Advanced Robotics (ICAR).

[29] LELY (2019). Lely Astronaut A4—Sistema Robotizado de Ordenha. 26p. https://www.lely.com/media/lely-centers-files/brochures/published/lely-astronaut_A4-PT.pdf.

[30] Zagidullin L.R., Khisamov R.R., Kayumov R.R., Shaidullin R.R., Zinnatov F.F., Sadykov N.F. (2023). Dairy robotic milking system. BIO Web Conf..

[31] Dohoo I.R., Leslie K.E. (1991). Evaluation of changes in somatic cell counts as indicators of new intramammary infections. Prev. Vet. Med..

[32] Fávero S., Portilho F.V.R., Oliveira A.C.R., Langoni H., Pantoja J.C.F. (2015). Factors associated with mastitis epidemiologic indexes, animal hygiene, and bulk milk bacterial concentrations in dairy herds housed on compost bedding. Livest. Sci..

[33] Garcia B.L.N., Martins C.M.M.R., Porto L.F., Nobrega D.B., Dos Santos M.V. (2024). Accuracy of an AI-based automated plate reading mobile application for the identification of clinical mastitis-causing pathogens in chromogenic culture media. Sci. Rep..

[34] Suojala L., Kaartinen L., Pyörälä S. (2013). Treatment for bovine *Escherichia coli* mastitis–an evidence-based approach. J. Vet. Pharmacol. Ther..

[35] Ruegg P.L. (2018). Making antibiotic treatment decisions for clinical mastitis. Vet. Clin. Food Anim..

[36] Fritz C.O., Morris P.E., Richler J.J. (2011). Effect size estimates: Current use, calculations, and interpretation. J. Exp. Psychol. Gen..

[37] Sharpe D. (2015). “Chi-Square Test is Statistically Significant: Now What?”. Pract. Assess. Res. Eval..

[38] SAS Institute, Inc. (2012). SAS OnDemand for Academics. Release 9.04.01M5P09132017.

[39] Costa J.C.M., Espeschit I.D.F., Pieri F.A., Benjamin L.A., Moreira M.A.S. (2014). Increase in biofilm formation by *Escherichia coli* under conditions that mimic the mastitic mammary gland. Cienc. Rural..

[40] Fidelis C.E., Orsi A.M., Freu G., Gonçalves J.L., Santos M.V.D. (2024). Biofilm Formation and Antimicrobial Resistance of *Staphylococcus aureus* and *Streptococcus uberis* Isolates from Bovine Mastitis. Vet. Sci..

[41] Petermann M., Wolter W., Rittershaus C., Kloppert B., Seufert H., Zschock M. Automatic milking systems: Udder health and milk flow profiles. Proceedings of the 1st North American Conference on Robotic Milking.

[42] Rotz C.A., Coiner C.U., Soder K.J. (2003). Automatic milking systems, farm size, and milk production. J. Dairy Sci..

[43] Campbell J.R., Marshall R.T. (2016). Dairy Production and Processing: The Science of Milk and Milk Products.

[44] Edmondson P. (2012). Mastitis control in robotic milking systems. In Pract..

[45] Pankey J.W. (1989). Premilking udder hygiene. J. Dairy Sci..

[46] Wieland M., Geary C.M., Nydam D.V., Virkler P.D., Zurakowski M., Watters R.D., Lynch R. (2023). A randomized controlled trial to study the effects of an automated premilking stimulation system on milking performance, teat tissue condition, and udder health in Holstein dairy cows. J. Dairy Sci..

[47] Hogenboom J.A., Pellegrino L., Sandrucci A., Rosi V., D’Incecco P. (2019). Invited review: Hygienic quality, composition, and technological performance of raw milk obtained by robotic milking of cows. J. Dairy Sci..

[48] Córdova H.A., Cardozo L.L., Alessio D.R.M., Thaler Neto A. (2018). Influence of udder depth on cleaning teats and health of the mammary gland in robotic milking. Arq. Bras. Med. Vet. Zootec..

[49] Cardozo L.L., Thaler Neto A., Souza G.N., Picinin L.C.A., Felipus N.C., Reche N.L.M., Schmidt F.A., Werncke D., Simon E.E. (2015). Risk factors for the occurrence of new and chronic cases of subclinical mastitis in dairy herds in southern Brazil. J. Dairy Sci..

[50] Hovinen M., Pyörälä S. (2011). Invited review: Udder health of dairy cows in automatic milking. J. Dairy Sci..

[51] Hogeveen H., Ouweltjes W., de Koning C.J.A.M., Stelwagen K. (2001). Milking interval, milk production and milk flow-rate in an automatic milking system. Livest. Prod. Sci..

[52] Edmondson P. (2012). Raised herd somatic cell count due to *Staphylococcus aureus* following the failure of an automatic teat spraying system. Vet. Rec..

[53] Mehrzad J., Duchateau L., Burvenich C. (2004). Viability of milk neutrophils and severity of bovine coliform mastitis. J. Dairy Sci..

[54] Munoz M.A., Bennett G.J., Ahlstrom C., Griffiths H.M., Schukken Y.H., Zadoks R.N. (2008). Cleanliness scores as indicator of *Klebsiella* exposure in dairy cows. J. Dairy Sci..

[55] Salfer J.A., Siewert J.M., Endres M.I. (2018). Housing, management characteristics, and factors associated with lameness, hock lesion, and hygiene of lactating dairy cattle on Upper Midwest United States dairy farms using automatic milking systems. J. Dairy Sci..

[56] Souza F.N., Cunha A.F., Rosa D.L., Brito M.A.V., Guimarães A.S., Mendonça L.C., Souza G.N., Lage A.P., Blagitz M.G., Della Libera A.M.M.P. (2016). Somatic cell count and mastitis pathogen detection in composite and single or duplicate quarter milk samples. Pesq. Vet. Bras..

[57] Svennersten-Sjaunja K.M., Pettersson G. (2008). Pros and cons of automatic milking in Europe. J. Anim. Sci..

[58] Mottram T. (1997). Requirements for teat inspection and cleaning in automatic milking systems. Comput. Electron. Agric..

[59] Taponen S., Liski E., Heikkilä A.M., Pyörälä S. (2017). Factors associated with intramammary infection in dairy cows caused by coagulase-negative *staphylococci*, *Staphylococcus aureus*, *Streptococcus uberis*, *Streptococcus dysgalactiae*, *Corynebacterium bovis*, or *Escherichia coli*. J. Dairy Sci..

[60] Janni K.A., Endres M.I., Reneau J.K., Schoper W. (2007). Compost Dairy Barn Layout and Management Recommendations. Appl. Eng. Agric..

[61] Nogara K.F., Busanello M., Tavares Q.G., De Assis J.A., Freu G., Dos Santos M.V., Vieira F.M.C., Zopollatto M. (2023). Factors influencing milk quality and subclinical mastitis in dairy herds housed in compost-bedded pack barn system. Animals.

[62] Nogara K.F., Busanello M., Haygert-Velho I.M.P., Zopollatto M., Frigeri K.D., Almeida P.S.G. (2021). Characterization and relationship between bulk tank milk composition and compost bedded variables from dairy barns in Rio Grande do Sul state, Brazil. Turk. J. Vet. Anim. Sci..

[63] Giambra I.J., Jahan Y., Yin T., Engel P., Weimann C., Brügemann K., König S. (2021). Identification of thermophilic aerobic sporeformers in bedding material of compost-bedded dairy cows using microbial and molecular methods. Animals.

[64] De Koning K., Slaghuis B., Van Der Vorst Y. (2003). Robotic milking and milk quality: Effects on bacterial counts, somatic cell counts, freezing point and free fatty acids. Ital. J. Anim. Sci..

[65] Dufour S., Fréchette A., Barkema H.W., Mussell A., Scholl D.T. (2011). Invited review: Effect of udder health management practices on herd somatic cell count. J. Dairy Sci..

[66] Vissio C., Mella A., Amestica L., Pol M. (2020). Noninferiority study evaluating the efficacy of a teat disinfectant containing copper and zinc for prevention of naturally occurring intramammary infections in an automatic milking system. J. Dairy Sci..

[67] Koivula M., Pitkälä A., Pyörälä S., Mäntysaari E.A. (2007). Distribution of bacteria and seasonal and regional effects in a new database for mastitis pathogens in Finland. Acta Agric. Scand..

[68] Barkema H.W., Schukken Y.H., Zadoks R.N. (2006). Invited review: The role of cow, pathogen, and treatment regimen in the therapeutic success of bovine *Staphylococcus aureus* mastitis. J. Dairy Sci..

